# How COVID-19 kick-started online learning in medical education—The DigiMed study

**DOI:** 10.1371/journal.pone.0257394

**Published:** 2021-09-21

**Authors:** Fabian Stoehr, Lukas Müller, Adrian Brady, Antoni Trilla, Aline Mähringer-Kunz, Felix Hahn, Christoph Düber, Nicole Becker, Marcus-Alexander Wörns, Julius Chapiro, Jan Bernd Hinrichs, Deniz Akata, Stephan Ellmann, Merel Huisman, David Koff, Sebastian Brinkmann, Fabian Bamberg, Oscar Zimmermann, Nikoleta I. Traikova, Jens U. Marquardt, D.-H. Chang, Fabian Rengier, Timo A. Auer, Tilman Emrich, Felix Muehler, Heinz Schmidberger, Bettina Baeßler, Daniel Pinto dos Santos, Roman Kloeckner

**Affiliations:** 1 Department of Diagnostic and Interventional Radiology, University Medical Center of the Johannes Gutenberg-University Mainz, Mainz, Germany; 2 Radiology Department, Mercy University Hospital, Cork, Ireland; 3 Department of Radiology, School of Medicine, University College Cork, Cork, Ireland; 4 Preventive Medicine and Epidemiology, Hospital Clínic of Barcelona Hospital, Barcelona, Spain; 5 Center for Quality Assurance and Development, Johannes Gutenberg-University Mainz, Mainz, Germany; 6 Department of Internal Medicine I, University Medical Center of the Johannes Gutenberg-University, Mainz, Germany; 7 Department of Radiology & Biomedical Imaging, Yale School of Medicine, New Haven, CT, United States of America; 8 Institute for Diagnostic and Interventional Radiology, Hannover Medical School, Hannover, Germany; 9 Department of Radiology, Hacettepe University Faculty of Medicine, Altındağ, Ankara, Turkey; 10 Institute of Radiology, University of Erlangen-Nuremberg, Erlangen, Germany; 11 Institute of Radiology, Meander Medical Center, Amersfoort, Utrecht, Netherlands; 12 Department of Radiology, McMaster University, Hamilton, ON, Canada; 13 Department of General, Visceral, Tumor and Transplantation Surgery, University Hospital Cologne, Cologne, Germany; 14 Department of Radiology, Medical Center—University of Freiburg, Freiburg, Germany; 15 University Hospital Cologne, Cologne, Germany; 16 Department of Radiology, Medical University of Plovdiv, Plovdiv, Bulgaria; 17 Department of Internal Medicine 1, University Hospital Schleswig-Holstein (UKSH), Lübeck, Germany; 18 Department of Radiology, Heidelberg University Hospital, Heidelberg, Germany; 19 Department of Radiology, Charité–Universitätsmedizin Berlin, corporate member of Freie Universität Berlin, Humboldt-Universität zu Berlin and Berlin Institute of Health, Berlin, Germany; 20 Division of Cardiovascular Imaging, Department of Radiology and Radiological Science, Medical University of South Carolina, Charleston, SC, United States of America; 21 German Centre for Cardiovascular Research, Partner site Rhine-Main, Mainz, Germany; 22 University Hospital Würzburg, Würzburg, Germany; 23 Department of Radiation Oncology, University Medical Center of the Johannes Gutenberg-University Mainz, Mainz, Germany; 24 Institute of Diagnostic and Interventional Radiology, University Hospital Zurich, Zurich, Switzerland; 25 Department of Radiology, University Hospital Cologne, Cologne, Germany; KTH Royal Institute of Technology, SWEDEN

## Abstract

**Background:**

The coronavirus disease 2019 (COVID-19) pandemic led to far-reaching restrictions of social and professional life, affecting societies all over the world. To contain the virus, medical schools had to restructure their curriculum by switching to online learning. However, only few medical schools had implemented such novel learning concepts. We aimed to evaluate students’ attitudes to online learning to provide a broad scientific basis to guide future development of medical education.

**Methods:**

Overall, 3286 medical students from 12 different countries participated in this cross-sectional, web-based study investigating various aspects of online learning in medical education. On a 7-point Likert scale, participants rated the online learning situation during the pandemic at their medical schools, technical and social aspects, and the current and future role of online learning in medical education.

**Results:**

The majority of medical schools managed the rapid switch to online learning (78%) and most students were satisfied with the quantity (67%) and quality (62%) of the courses. Online learning provided greater flexibility (84%) and led to unchanged or even higher attendance of courses (70%). Possible downsides included motivational problems (42%), insufficient possibilities for interaction with fellow students (67%) and thus the risk of social isolation (64%). The vast majority felt comfortable using the software solutions (80%). Most were convinced that medical education lags behind current capabilities regarding online learning (78%) and estimated the proportion of online learning before the pandemic at only 14%. In order to improve the current curriculum, they wish for a more balanced ratio with at least 40% of online teaching compared to on-site teaching.

**Conclusion:**

This study demonstrates the positive attitude of medical students towards online learning. Furthermore, it reveals a considerable discrepancy between what students demand and what the curriculum offers. Thus, the COVID-19 pandemic might be the long-awaited catalyst for a new “online era” in medical education.

## 1. Background

Since early 2020, the whole world has been in a state of suspension of normal activity due to the coronavirus disease 2019 (COVID-19) pandemic. Almost all countries have been heavily impacted by the outbreak [1]. With the aim of stopping the spread of the virus, far-reaching restrictions of social and professional life have been adopted [2]. Medical schools had to restructure their curriculum by switching from on-site teaching to online teaching [3–6]. Because of the rapid progress of the pandemic, these changes had to be implemented under considerable time pressure.

Today’s students are highly interested in innovative teaching methods including online learning, networked learning, simulation-based learning and others [7–9]. However, as medical teaching is mainly based on traditional, ex-cathedra concepts, only a minority of medical schools had implemented such innovative concepts prior to COVID-19 [10–12]. One frequent argument against online learning in the past was that its implementation is a time-intensive and expensive process, due to the lack of the infrastructure needed [8, 13].

However, in the wake of the pandemic, such teaching concepts have been successfully introduced to a much greater extent in recent months [3–6]. However, it can be assumed that quickly switching to fully online teaching led to “emergency remote teaching” instead of a structured, dedicated online teaching curriculum. Although, the current situation might present a unique opportunity for the modernization of medical education in order to fulfill students’ teaching needs, opinions still differ on how medical education should be delivered in the future.

Thus, we aimed to describe the current status of teaching in medical education by evaluating medical students’ attitudes towards online learning in general, the “real life” implementation of online learning in medical schools during the pandemic and possible teaching scenarios after the pandemic. We decided to conduct an international, multicenter study in order to provide a broad, scientific basis to guide future development of medical education.

## 2. Methods

### 2.1 Study set-up

This survey was conducted as a cross-sectional study including several medical schools in 12 different countries (Bulgaria, Canada, Greece, Germany, Hungary, Ireland, Spain, Switzerland, the Netherlands, Turkey, Ukraine, USA). As this survey was addressed to medical students, this study is based on a non-probability, voluntary sample. We followed the recommendations of the EQUATOR network (“https://www.equator-network.org”) and conducted this web-based, cross-sectional study in accordance with the “Checklist for Reporting Results of Internet E-Surveys (**CHERRIES**)” [14] and with the “Strengthening the Reporting of Observational Studies in Epidemiology (**STROBE**) Statement: guidelines for reporting observational studies” [15] (S1 Checklist). Institutional review board approval was waived by the Ethics Committee of the Medical Association of Rhineland-Palatinate. The study design was conducted in accordance to the Declaration of Helsinki. The survey started on June 12, 2020 and was closed on August 7, 2020.

### 2.2 Questionnaire design

A dedicated questionnaire was designed together with the Center for Quality Assurance and Development (ZQ) of the Johannes Gutenberg University Mainz. The questionnaire consisted of various sections covering, in particular, the following aspects: Current online learning situation at medical schools, types of online learning currently offered and desired in future, technical and social aspects of online learning, attitudes towards the current and future role of online learning in medical education, suitability of teaching concepts for online learning, as well as current and desired ratio between online and on-site learning. In total, the questionnaire comprised 35 questions. Students were asked to answer the questions using a 7-point Likert scale (1 = “strongly disagree,” 2  =  “disagree,” 3  =  “somewhat disagree,” 4  =  “neutral,” 5  =  “somewhat agree,” 6 = “agree,” to 7 = “strongly agree”). We deliberately chose a 7-point scaling system for the following reasons: compared to a 5-point scale, a 7-point scale leads to a higher variance of answers and thus to higher reliability and more nuanced trend analysis [16]; scaling up higher to 9-point or even 11-point systems, however, would not yield added value regarding the information obtained and would have strained the abstraction capabilities of our respondents [17]. The entire questionnaire is attached in the supplement (S1 Questionnaire).

### 2.3 Validation of the questionnaire

The questionnaire underwent a two-step external validation approach in order to further enhance the quality of the study. First, cognitive pretesting was performed on a small sample size of 10 students. First, cognitive pretesting was performed on a small sample size of 10 students [18]; second, pilot testing was performed on a larger cohort of 50 medical students from two university medical centers [19]. The final questionnaire was transferred to an established online survey tool (SurveyMonkey®, www.surveymonkey.com), which was also used for data collection.

### 2.4 Distribution of the questionnaire

Invitations to take part in the study were sent via email from the Deans’ offices and/or via social media to all students of the participating medical schools. The invitations were further distributed by the German (BVMD) and the European Medical Students’ Association (EMSA). All invitations contained an identical short introductory text and the hyperlink to access the survey. The students were informed that the survey results would be anonymous and that they were collected for research purposes only.

### 2.5 Statistical analysis

Final survey results were extracted as CSV file and subsequently analyzed using R 4.0.2 (A Language and Environment for Statistical Computing, R Foundation for Statistical Computing, https://www.R-project.org; accessed July 2020). Figures were plotted using the ggplot2 and likert packages [20, 21]. Mean and standard deviation were calculated to analyze results [22]. Comparison between responses was performed using Welch’s t-test [23, 24]. A p-value <0.05 was considered statistically significant. For further subgroup analysis regarding sex and study year, Cohen’s d was calculated; effect size was considered large if >0.5.

## 3. Results

### 3.1 Participants’ demographics

**Fig 1:**
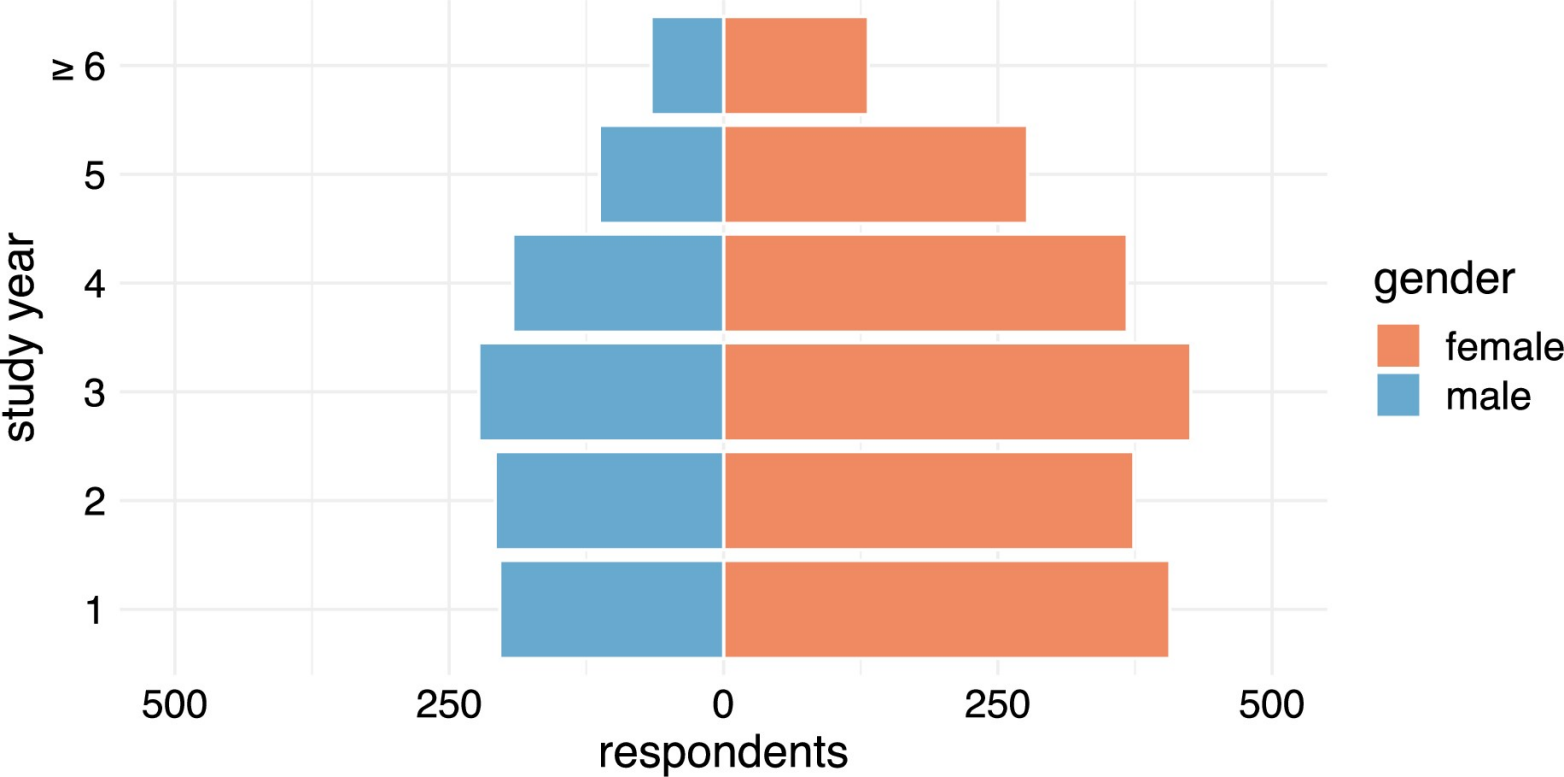
Distribution of the participants according to sex and to their current study year. A total of 29 participants are not included in this figure due to: non-binary gender (n = 16), missing gender (n = 9), and missing study year (n = 4).

A total of 3286 participants completed the questionnaire. The demographic data recorded were sex (33% male, 66.2% female, 0.5% non-binary), age (mean 23.6 years), and current study year (mean 3rd study year) (Table 1). The detailed distribution of the participants according to study year and gender can be found in Fig 1.

**Table 1:** Demographic characteristics of the participants.

**Sex** ^**1**^	
Female n (%)	2177 (66.2%)
Male n (%)	1084 (33%)
Non-binary n (%)	16 (0.5%)
**Age** ^**2**^	
Mean y (SD)	23.6 (4.0)
**Study year** ^**3**^	
Mean y (SD)	3 (1.3)

SD = standard deviation.

^1^Sex missing in 9/3286 questionnaires (0.3%).

^2^Age missing in 37/3286 questionnaires (1.1%).

^3^Study year missing in 4/3286 questionnaires (0.1%).

### 3.2 Survey results

**Fig 2:**
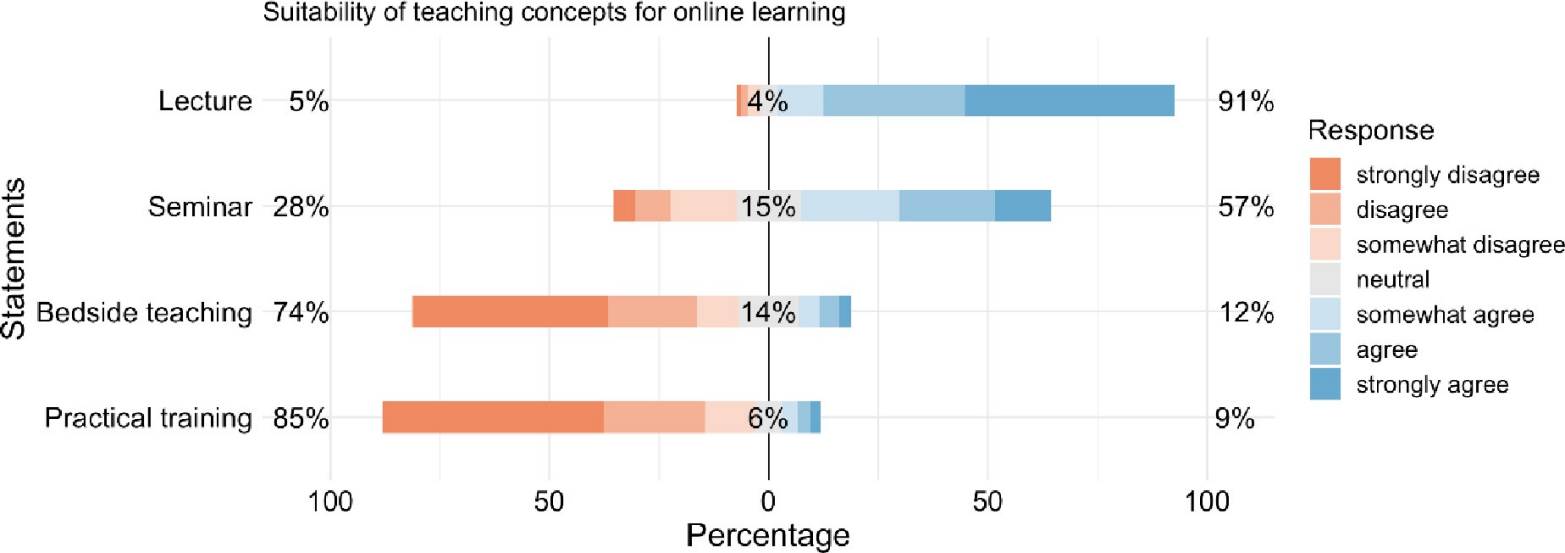
Centered stacked bar plot showing the detailed responses of the participants regarding the “suitability of teaching concepts for online learning”. Ranking ranges from highly suitable (lecture) to unsuitable (practical training).

**Fig 3:**
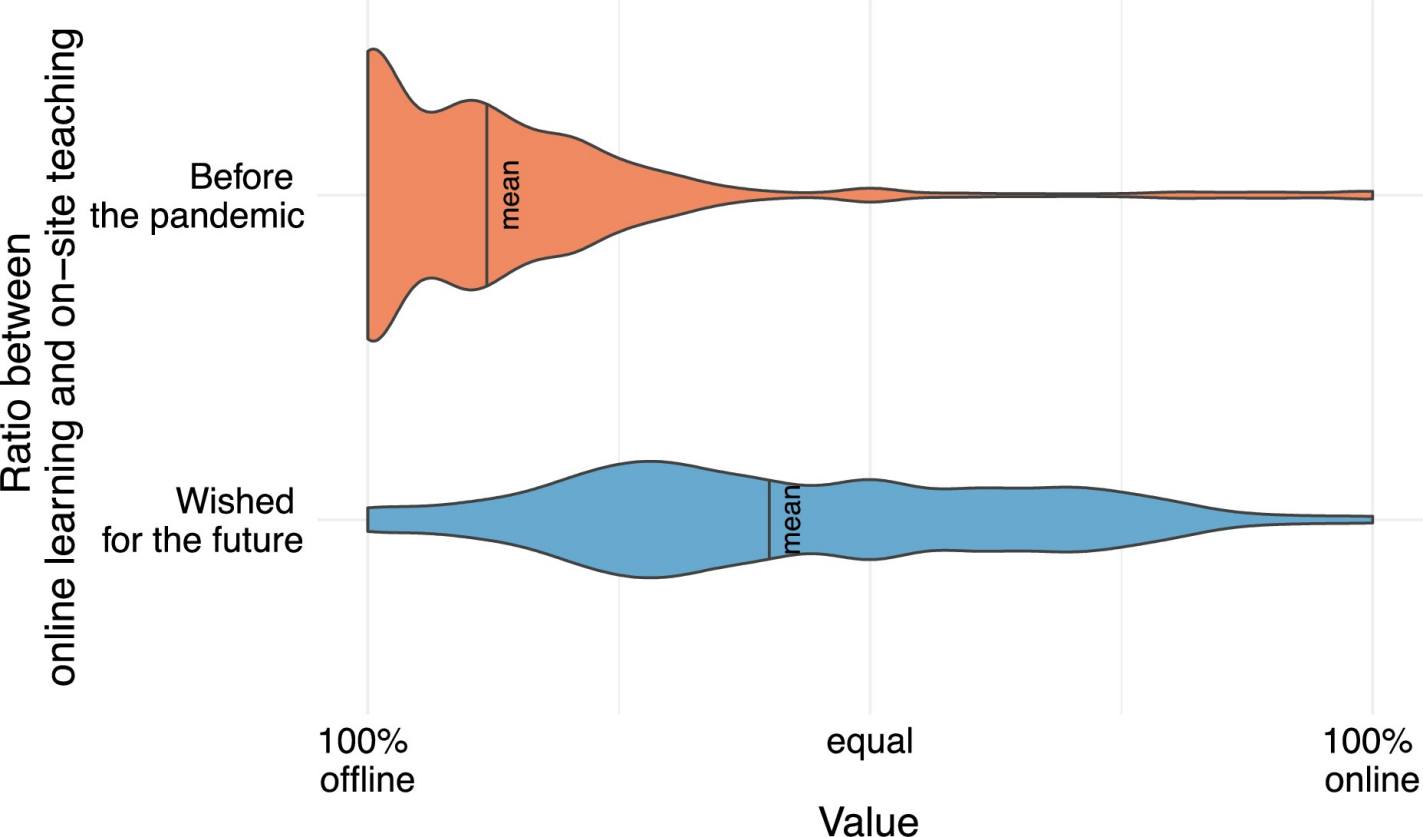
The upper violin plot depicts the ratio between online learning and on-site learning before the pandemic (mean 14%). The lower violin plot depicts the ratio between online learning and on-site learning wished for the future (mean 42%).

**Fig 4:**
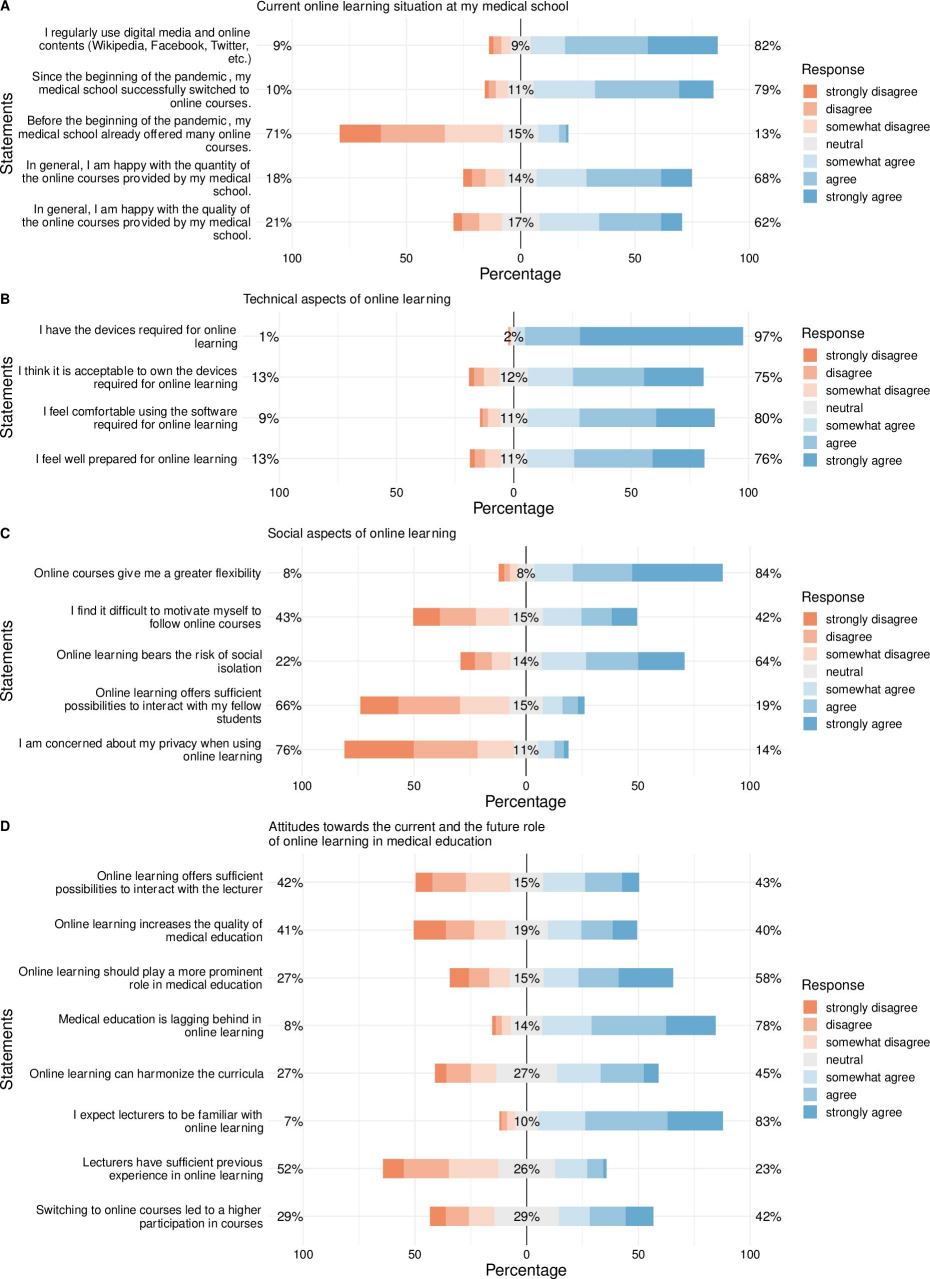
Centered stacked bar plot showing the detailed responses of the participants regarding the “current online learning situation” (A), the “technical aspects of online learning” (B), the “social aspects of online learning” (C), and the “attitudes towards the current and the future role of online learning in medical education” (D). Orange tones represent “disagreement”, grey represents “neutral”, and blue tones represent “agreement”.

In the following, survey results for each particular category are presented in written form as well as graphically (Figs 2–4 and S1 Fig). To increase comprehensibility, the text contains only the key findings and summarizes “strongly disagree”, “disagree” and “somewhat disagree” as disagreement and “somewhat agree”, “agree” and “strongly agree” as agreement. For statistical analyses, the original categories were used. All detailed data including the particular results per item, particular response rates per item and means per item are provided in the supplementary material (S1–S4 Tables). The entire results are presented visually in Fig 4.

#### 3.2.1 Current online learning situation at medical school

The vast majority of medical schools successfully switched to online learning (78%), although only very few (13%) offered many online courses before the pandemic. Most students were satisfied with the quantity and quality of the online courses offered by their medical schools during the pandemic (67% and 62%, respectively). To capture the participants’ comfort with online activity, they were asked about the extent of their social media use, which are frequently used by a vast majority (82%) (Fig 4 and S1 Table).

#### 3.2.2 Types of online learning currently offered and desired in future

Pre-recorded lectures or seminars without the possibility of interaction (88%) and interactive live lectures or seminars (76%) were by far the most common types of online learning offered by medical schools. Online platforms and resources for self-learning/self-assessment modules were also common (70%). Chats (with/without video stream) (40%) were less frequently offered, and audio podcasts (13%) were uncommon. The ranking of the participants’ preferences regarding their desired types of online learning in future followed exactly the same order (S1 Fig).

#### 3.2.3 Technical aspects of online learning

The great majority of students already owned the electronic devices required for online learning (97%) and was further willing to invest in such devices (74%). Most participants preferred a computer (79%), fewer preferred a tablet (20%), and only very few used a smartphone (1%). Most participants felt comfortable using the software solutions for online learning (80%). All in all, three-quarters of the participants felt well prepared for online learning (76%) (Fig 4 and S2 Table).

#### 3.2.4 Social aspects of online learning

Online learning provided increased flexibility (84%). However, a considerable proportion had difficulties in motivating themselves to follow online learning modules (42%). For around two-thirds, online learning is perceived as creating the risk of social isolation (64%) and does not offer sufficient possibilities for interaction with fellow students (67%). Very few are concerned about their privacy when using online learning (14%) (Fig 4 and S3 Table).

#### 3.2.5 Attitudes towards the current and future role of online learning in medical education

The vast majority believes that medical education in general lags behind current capabilities regarding online learning (78%). Thus, few participants were satisfied with the current role of online learning in medical education (27%). Only 22% considered the previous experience of teachers in online learning to be sufficient but, in contrast, a vast majority expects them to be familiar with online teaching (83%). For around three-quarters of the participants, switching to online learning led to a higher or unchanged attendance of courses (70%) (Fig 4 and S4 Table).

#### 3.2.6 Suitability of teaching concepts for online learning

The participants´ rating regarding the suitability of online learning for different teaching concepts is as follows: 1. lecture: (91%); 2. seminar: (57%); 3. bedside teaching: (12%); 4. practical training: (9%) (Fig 2).

#### 3.2.7 Current and desired ratio between online and on-site learning

In the last two questions, students were asked to estimate the ratio between online learning and on-site teaching before the pandemic and to state the ratio desired for the future. Before the pandemic, students estimated the ratio between online learning and on-site teaching at 14% online vs. 86% on-site teaching. In future, participants wish for a more balanced ratio of 42% online teaching and 58% on-site teaching (p<0.05); however, the distribution shows considerable variability between 25% and 75% online teaching. The detailed distribution is provided in a violin plot (Fig 3).

#### 3.2.8 Subgroup analyses regarding gender and study year as well as country of studies

Further subgroup analyses according to gender and study year were performed. According to effect sizes, neither gender nor study year had a significant impact on the abovementioned results. The detailed statistical analyses can be found in the supplementary material (S5 and S6 Tables).

## 4. Discussion

With more than 3,000 participants from countries with a wide geographical distribution, this study about online learning in medical education underlines the immense relevance of this topic for medical students and for medical education in general. In summary, our study suggests the positive attitude of an overwhelming majority of medical students towards online learning. Furthermore, students are convinced that medical schools have heretofore neglected digitization of learning and that medical education is lagging behind its capabilities. Consistent with that, medical students estimate the pre-pandemic proportion of online learning in medical education at only 14%. In future, students wish for a more balanced ratio with at least 40% of online learning compared to on-site teaching.

Before the pandemic, teaching in most medical schools has been mainly based on “traditional” concepts requiring physical presence of the students [11, 13, 25, 26]. The outbreak of the COVID-19 pandemic led to a sudden switch from on-site teaching to almost entirely online teaching [3–6]. However, this switch was mostly born out of necessity and might have caused problems in the teaching “logistics” of medical schools. Technical infrastructure had to be installed and teachers had to adapt their courses immediately. However, our survey results point out that students were nevertheless satisfied with both quality and quantity of the online courses provided. This means that most medical schools were able to master the change from on-site to online teaching at short notice. This refutes the widespread opinion that switching to online learning is both a time-intensive and expensive process due to the lack of the infrastructure needed [8, 13, 27]. To summarize, our results indicate that there might be more infrastructure available for online teaching in medical schools than initially expected.

Despite this successful switch to online learning, however, downsides of online learning should be considered. One issue is the possible danger of social isolation because of reduced “real” discussions and less interaction with fellow students or teachers [7, 28]. This also includes a lack of distinction between home and “workplace” due to increased study times at home [29]. Our results suggest that the participants took this issue seriously. However, social isolation may be aggravated in view of the concurrent situation of a mandatory nationwide quarantine in many countries. Thus, it might be assumed that after the pandemic, social life will take place again.

Furthermore, our results indicate that a considerable proportion of participants had difficulties in motivating themselves to follow online learning modules. However, this seems to be a multifactorial issue and recent studies on students’ motivation suggest that the major “motivational” factor may be the teaching type (e.g. problem-based vs. lecture-based) and not the teaching format (e.g. online vs. on-site teaching) [30, 31]. Because of the rapid switch to online learning, most medical schools did not have a specific curriculum framework of online learning. Thus, possible solutions for the future could be to not only provide single online courses but to integrate these into a dedicated framework including a schedule for each subject and frequent performance reviews, to avoid leaving students behind.

However, even before the pandemic, declining attendance rates of students have become part of everyday medical school life [32, 33]. Various possible reasons have been discussed [32, 33]; and, as our results indicate, change of students’ learning mentalities and environment seem to be major factors. For example, access to broad online learning opportunities without the necessity for any physical presence is very easy nowadays. Our results show that medical students often use online content and appreciate various types of online learning. Furthermore, most students are willing to invest in the electronic devices required for online learning, which implies their high intrinsic motivation. With this in mind, online teaching could be an answer to the needs of an increasingly diverse student community [34]. As online learning provides increased flexibility, it could be crucial in terms of accommodating student diversity and inclusivity.

Despite this obviously strong demand for online learning among medical students, there are parts of medical education, which seem unsuitable for online learning. For example, medical students as doctors-to-be require live patient contact via face-to-face interaction to develop clinical skills. The participants of our study recognize this as an important point, and judge digitization in medical education in a highly differentiated way. The students deem lectures and seminars but not bedside teaching and practical skills training appropriate for online teaching. Nonetheless, regarding elements such as practical skills training, many options exist for using innovative teaching concepts as a “dry run” before facing real patients [35, 36]. This is in line with several studies underpinning the positive effect of laparoscopic, arthroscopic and endovascular simulator training which increases not only hands-on skills but also can stimulate interest in the particular subspecialty [37–39]. Furthermore, virtual reality technologies including e.g. real-time holoportation and smartglasses are advancing rapidly, and allow for a virtual “real life” experience [40]. There are already promising results showing the successful use of those innovative technologies in clinical education [40, 41].

Regarding the results of this study, the overall challenge is to reasonably implement innovative online learning concepts in the curriculum. Although the shift towards online learning started as “emergency remote teaching” instead of a structured transition, several innovative concepts had been implemented during the last few months. Nevertheless, the participants in our study are convinced that the current curriculum can be enriched by the use of online teaching methods. Thus, as a benchmark for the future, students deem a ratio of around 42% online and 58% on-site teaching ideal.

In order to improve current medical education sustainably, a primary goal should be the implementation of a curriculum framework of online learning. Having the desired ratio of online and on-site teaching in mind, With this in mind, “blended learning”, which combines on-site and online learning as a “hybrid”, could be a good model [42, 43]. In order to actively enhance the learning process, students would have to prepare the teaching content independently using online resources before a dedicated, problem-centered on-site course is delivered [42, 44, 45].

### 4.1 Limitations

Our study is a questionnaire-based survey set up on a non-probability, voluntary sample and therefore entails typical pitfalls of those. We had to consider selection bias, meaning that interested students are more likely to complete the questionnaire [46]. This might particularly apply to those students who were included via social media, which we did not record due to the anonymous study design. However, we had a very high number of participants from many different medical schools in many countries, suggesting a well-mixed pool of participants. Due to our study design, we could only calculate completion rate, since no information was available on the total number of potentially contacted participants. Furthermore, there is the potential for social desirability, meaning that participants choose the answer that they assume is favourable [47]. To attenuate this kind of response bias, we created a completely anonymous and untraceable study design and instructed the students that the survey results were for research purposes only. Furthermore, we only investigated participants from industrialized countries across Europe and North America, and results could be entirely different if the survey would have been distributed in e.g. emerging or developing countries. Further studies could include those countries in order to gain deeper insights regarding socio-economic dimensions of teaching in medical education.

Ultimately, we decided on a cross-sectional study design, which allows only for a snapshot. Thus, as a limitation, our results may not necessarily translate into long-term opinions informing students’ perception of online learning. To assess these effects, further longitudinal studies are necessary. Such follow-up studies could e.g. focus on the purpose of teaching and which concepts or combinations are most suitable to impart knowledge to medical students. This could provide a more in-depth analysis of different teaching types. Furthermore, we primarily focused on students’ attitudes towards online teaching. Thus, further research focusing on effectiveness and markers of mental health may be needed to better understand the impact of distance learning on students. Further studies could also investigate teachers’ attitudes towards online teaching as well as potential differences in how both groups see “their” future of medical education.

## 5. Conclusion

In summary, there is an obvious discrepancy between what medical students desire and what current curricula offer them. Therefore, it is our duty to restructure medical education not only for the present but even more so for the post-pandemic future in a sustainable manner. As medical students clearly see potential for improvement regarding the online teaching skills of their lecturers, medical faculties should put further effort in imparting these skills to lecturers.

Positively speaking, the COVID-19 pandemic might be the long-awaited and much-needed catalyst for a new online teaching era in medical education. Furthermore, the current situation gives students as well as teachers a unique opportunity to create and further advance innovative learning and teaching concepts together. The medical community seems to understand the need for change and has already started the process.

## Supporting information

S1 Checklist Guidelines for reporting observational studies. (PDF)

S1 Questionnaire Questionnaire investigating various aspects of online learning in medical education. (PDF)

S1 Fig Ranking of the participants’ preferences regarding their desired types of online learning currently offered and in future. Boxplots showing the proportion of types of online learning currently offered (A) and the ranking of types of online learning desired in future by the students from top (most desired) to bottom (least desired) (B).
(PDF)

S1 Table Current online learning situation at medical school (n = 3286). SD = standard deviation; N/A = not available.
(PDF)

S2 Table Technical aspects of online learning school (n = 3286). SD = standard deviation; N/A = not available.
(PDF)

S3 Table Social aspects of online learning (n = 3286). SD = standard deviation; N/A = not available.
(PDF)

S4 Table Attitudes towards the current and the future role of online learning in medical education (n = 3286). SD = standard deviation; N/A = not available.
(PDF)

S5 Table Subgroup analysis regarding study year. (PDF)

S6 Table Subgroup analysis regarding gender. (PDF)
