# Supplementary material for: How COVID-19 kick-started online learning in medical education—The DigiMed study
Source: PLoS One. 2021 Sep 21;16(9):e0257394. doi: 10.1371/journal.pone.0257394 (PMC8454930; doi:10.1371/journal.pone.0257394)
Supplement: S1 Questionnaire — (PDF) [file pone.0257394.s002.pdf]

1. Gender

- ◯ Male
- ◯ Female
- ◯ Other

2. Age

3. Current study year

4. Country where I study in

5. Social media:

	1 (strongly disagree)	2 (disagree)	3 (somewhat disagree)	4 (neutral)	5 (somewhat agree)	6 (agree)	7 (strongly agree)
**I regularly use digital media** and online contents (Wikipedia, Facebook, Twitter, etc.)	◯	◯	◯	◯	◯	◯	◯

6. **Online learning during the COVID-19 pandemic:**

	1 (strongly disagree)	2 (disagree)	3 (somewhat disagree)	4 (neutral)	5 (somewhat agree)	6 (agree)	7 (strongly agree)
**Since** the beginning of the pandemic, my medical school **successfully switched** to online courses.	○	○	○	○	○	○	○
**Before** the beginning of the pandemic, my medical school **already offered** many online courses.	○	○	○	○	○	○	○
In general, I am happy with the **quantity** of the online courses provided by my medical school.	○	○	○	○	○	○	○
In general, I am happy with the **quality** of the online courses provided by my medical school.	○	○	○	○	○	○	○

7. The following types of online learning are offered by my medical school (multiple answers possible):

- [ ] Interactive live lectures or seminars (webinars)
- [ ] pre-recorded lectures or seminars without possibility of interaction
- [ ] Online platforms and ressources for self-learning/self-assessment modules
- [ ] Chats (with/without video stream)
- [ ] Audio podcasts

8. In future, I wish the following types of online learning methods will be offered by my medical school. Please rank via "drop down" from 1-5 (1=most preferred, 5=least preferred):

Interactive live lectures or seminars (webinars)

Pre-recorded lectures or seminars without possibility of interaction

Online platforms and resources for self-learning/self-assessment modules.

Chats (with/without video stream)

Audio podcasts

9. Technical aspects of online learning:

	1 (strongly disagree)	2 (disagree)	3 (somewhat disagree)	4 (neutral)	5 (somewhat agree)	6 (agree)	7 (strongly agree)
I have the **devices available** required for online learning (computer, tablet, smartphone, etc).	○	○	○	○	○	○	○
I think it is **acceptable to invest** in electronic devices required for online learning (computer, tablet, etc.).	○	○	○	○	○	○	○
I feel **comfortable using the software** solutions used for online learning (e.g. Microsoft Teams, Moodle, Google Hangout, Zoom, GoTo-Meeting, Skype, etc.).	○	○	○	○	○	○	○
In general, I feel **well prepared** for online learning.	○	○	○	○	○	○	○

10. I prefer the following device for online learning:

11. Online learning in medical education:

	1 (strongly disagree)	2 (disagree)	3 (somewhat disagree)	4 (neutral)	5 (somewhat agree)	6 (agree)	7 (strongly agree)
Online learning offers sufficient possibilities to **interact** with the **lecturers.**	○	○	○	○	○	○	○
Online learning increases the **quality of medical education** in general.	○	○	○	○	○	○	○
Online learning should play a **more prominent role** in medical education even beyond the pandemic.	○	○	○	○	○	○	○

12. My involvement with online learning:

	1 (strongly disagree)	2 (disagree)	3 (somewhat disagree)	4 (neutral)	5 (somewhat agree)	6 (agree)	7 (strongly agree)
I like that online courses give me a **greater flexibility** in planning my day and the time I spend on studying.							
I find it **difficult to motivate** myself to follow online courses.							
I think online learning bears the risk of **increased social isolation.**							
Online learning offers sufficient possibilities to interact with my **fellow students.**							
I am concerned about **my privacy** when using online learning materials.							

13. The future of online learning:

	1 (strongly disagree)	2 (disagree)	3 (somewhat disagree)	4 (neutral)	5 (somewhat agree)	6 (agree)	7 (strongly agree)
Medical education is **lagging behind** in online learning.							
Online learning offers the chance to **harmonize** the **curricula** between medical schools.							
Nowadays, I expect **lecturers to be familiar** with online learning.							
**Lecturers** have **sufficient previous experience** in online learning.							

14. Switching to online learning

	1 (much less)	2 (less)	3 (a bit less)	4 (neutral)	5 (a bit higher)	6 (higher)	7 (much higher)
Regarding yourself, did switching to online learning lead to a **higher or a lower participation** in courses?							

15. How much are the following teaching concepts suitable for online learning?

	1 (completely unsuitable)	2 (unsuitable)	3 (a bit unsuitable)	4 (neutral)	5 (a bit suitable)	6 (suitable)	7 (highly suitable)
Lecture	◯	◯	◯	◯	◯	◯	◯
Seminar	◯	◯	◯	◯	◯	◯	◯
Bedside teaching	◯	◯	◯	◯	◯	◯	◯
Practical training, e.g. simulator training	◯	◯	◯	◯	◯	◯	◯

16. <u>Before the pandemic</u>, the mix between offline and online learning was:

On-site (100%) Equal mix (50%/50%) Online (100%)

17. <u>In future</u>, the <u>perfect</u> mix between offline and online learning is:

On-site (100%) Equal mix (50%/50%) Online (100%)

Thank you very much for your participation!

The DigiMEd 2020 team
