# Supplementary material for: How COVID-19 kick-started online learning in medical education—The DigiMed study
Source: PLoS One. 2021 Sep 21;16(9):e0257394. doi: 10.1371/journal.pone.0257394 (PMC8454930; doi:10.1371/journal.pone.0257394)

S3 Figure. Ranking of the participants' preferences regarding their desired types of online learning currently offered and in future

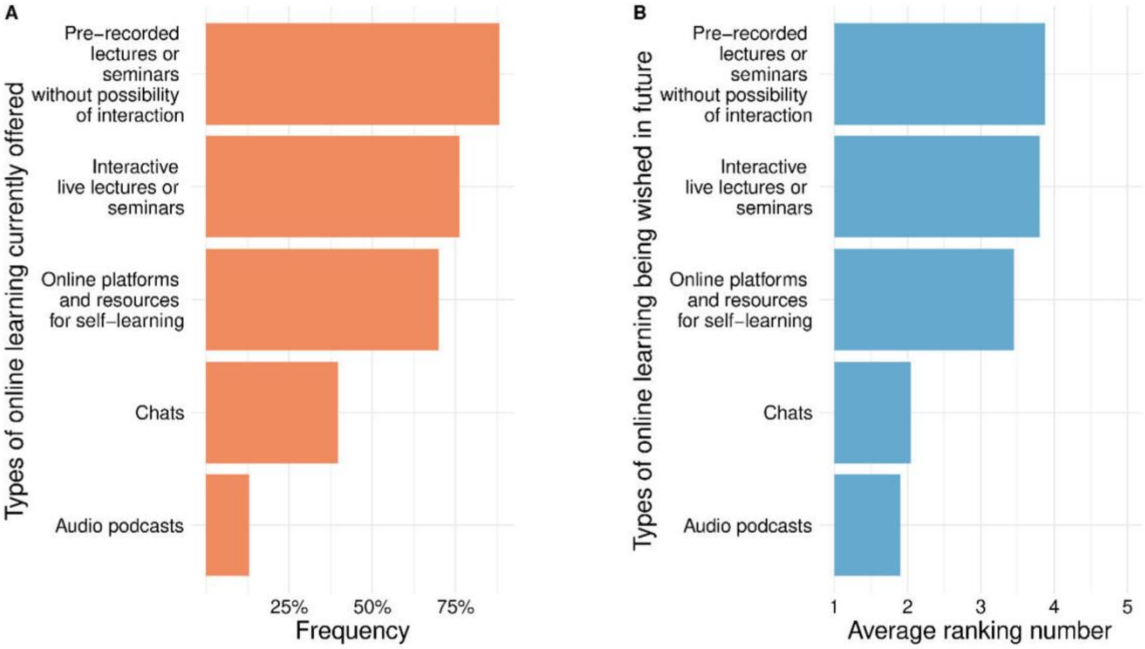

Supplement: S1 Fig — Boxplots showing the proportion of types of online learning currently offered (A) and the ranking of types of online learning desired in future by the students from top (most desired) to bottom (least desired) (B). (PDF) [file pone.0257394.s003.pdf]
