# Supplementary material for: How COVID-19 kick-started online learning in medical education—The DigiMed study
Source: PLoS One. 2021 Sep 21;16(9):e0257394. doi: 10.1371/journal.pone.0257394 (PMC8454930; doi:10.1371/journal.pone.0257394)
Supplement: S1 Table — SD = standard deviation; N/A = not available. (PDF) [file pone.0257394.s004.pdf]

S4 Table. Current online learning situation at medical school (n= 3286)

Statement	Strongly disagree n (%)	Disagree n (%)	Somewhat disagree n (%)	Neutral n (%)	Somewhat agree n (%)	Agree n (%)	Strongly Agree n (%)	N/A n (%)	Mean ± SD
Since the pandemic, my medical school successfully switched to online courses	53 (1.6%)	104 (3.2%)	174 (5.3%)	381 (11.6%)	889 (27.1%)	1207 (36.7%)	470 (14.3%)	8 (0.2%)	5.3 ± 1.3
Before the pandemic, my medical school already offered many online courses	599 (18.2%)	924 (28.1%)	822 (25.0%)	477 (14.5%)	290 (8.8%)	96 (2.9%)	31 (0.9%)	47 (1.4%)	2.8 ± 1.4
I am happy with the quantity of online courses provided	122 (3.7%)	205 (6.2%)	282 (8.6%)	458 (13.9%)	726 (22.1%)	1054 (32.1%)	426 (13.0%)	13 (0.4%)	4.9 ± 1.6
I am happy with the quality of online courses provided	126 (3.8%)	248 (7.5%)	332 (10.1%)	548 (16.7%)	870 (26.5%)	862 (26.2%)	288 (8.8%)	12 (0.4%)	4.7 ± 1.6
I regularly use social media	66 (2.0%)	114 (3.5%)	130 (4.0%)	290 (8.8%)	505 (15.4%)	1178 (35.8%)	99 (30.3%)	8 (0.2%)	5.6 ± 1.4

SD=standard deviation; N/A=not available
