# Supplementary material for: How COVID-19 kick-started online learning in medical education—The DigiMed study
Source: PLoS One. 2021 Sep 21;16(9):e0257394. doi: 10.1371/journal.pone.0257394 (PMC8454930; doi:10.1371/journal.pone.0257394)
Supplement: S2 Table — SD = standard deviation; N/A = not available. (PDF) [file pone.0257394.s005.pdf]

S5 Table. Technical aspects of online learning school (n= 3286)

Statement	Strongly disagree n (%)	Disagree n (%)	Somewhat disagree n (%)	Neutral n (%)	Somewhat agree n (%)	Agree n (%)	Strongly agree n (%)	N/A n (%)	Mean ± SD
I have the devices required for online learning	12 (0.4%)	10 (0.3%)	19 (0.6%)	61 (1.9%)	121 (3.7%)	768 (23.4%)	2288 (69.6%)	7 (0.2%)	6.6 ± 0.8
I think it is acceptable to own the devices required for online learning	76 (2.3%)	139 (4.2%)	224 (6.8%)	409 (12.4%)	627 (19.1%)	986 (30.0%)	819 (24.9%)	6 (0.2%)	5.3 ± 1.5
I feel comfortable using the software required for online learning	33 (1.0%)	81 (2.5%)	177 (5.4%)	372 (11.3%)	724 (22.0%)	1070 (32.6%)	819 (24.9%)	10 (0.3%)	5.5 ± 1.3
I feel well prepared for online learning	65 (2.0%)	137 (4.2%)	225 (6.8%)	365 (11.1%)	670 (20.4%)	1089 (33.1%)	726 (22.1%)	9 (0.3%)	5.3 ± 1.5

SD=standard deviation; N/A=not available
