# Supplementary material for: How COVID-19 kick-started online learning in medical education—The DigiMed study
Source: PLoS One. 2021 Sep 21;16(9):e0257394. doi: 10.1371/journal.pone.0257394 (PMC8454930; doi:10.1371/journal.pone.0257394)
Supplement: S3 Table — SD = standard deviation; N/A = not available. (PDF) [file pone.0257394.s006.pdf]

S6 Table. Social aspects of online learning (n= 3286)

Statement	Strongly Disagree n (%)	Disagree n (%)	Somewhat disagree n (%)	Neutral n (%)	Somewhat agree n (%)	Agree n (%)	Strongly agree n (%)	N/A n (%)	Mean ± SD
Online courses give me a greater flexibility	78 (2.4%)	86 (2.6%)	120 (3.7%)	246 (7.5%)	563 (17.1%)	875 (26.6%)	1312 (39.9%)	6 (0.2%)	5.7 ± 1.5
I find it difficult to motivate myself to follow online courses	381 (11.6%)	523 (15.9%)	486 (14.8%)	504 (15.3%)	563 (17.1%)	462 (14.1%)	362 (11.0%)	5 (0.2%)	4.0 ± 1.9
Online learning bears the risk of social isolation	205 (6.2%)	239 (7.3%)	264 (8.0%)	455 (13.8%)	655 (19.9%)	776 (23.6%)	683 (20.8%)	9 (0.3%)	4.9 ± 1.8
Online learning offers sufficient possibilities to interact with my fellow students	553 (16,8%)	913 (27.8%)	722 (22.0%)	489 (14.9%)	286 (8.7%)	218 (6.6%)	95 (2.9%)	10 (0.3%)	3.0 ± 1.6
I am concerned about my privacy when using online learning	994 (30.2%)	938 (28.5%)	537 (16.3%)	360 (11.0%)	236 (7.2%)	142 (4.3%)	70 (2.1%)	9 (0.3%)	2.6 ± 1.6

SD=standard deviation; N/A=not available
