# Supplementary material for: How COVID-19 kick-started online learning in medical education—The DigiMed study
Source: PLoS One. 2021 Sep 21;16(9):e0257394. doi: 10.1371/journal.pone.0257394 (PMC8454930; doi:10.1371/journal.pone.0257394)
Supplement: S4 Table — SD = standard deviation; N/A = not available. (PDF) [file pone.0257394.s007.pdf]

S7 Table. Attitudes towards the current and the future role of online learning in medical education (n= 3286)

Statement	Strongly disagree n (%)	Disagree n (%)	Somewhat disagree n (%)	Neutral n (%)	Somewhat agree n (%)	Agree n (%)	Strongly agree n (%)	N/A n (%)	Mean ± SD
Online learning offers sufficient possibilities to interact with the lecturer	252 (7.7%)	490 (14.9%)	651 (19.8%)	494 (15.0%)	615 (18.7%)	534 (16.3%)	241 (7.3%)	9 (0.3%)	4.0 ± 1.7
Online learning increases the quality of medical education	475 (14.5%)	420 (12.8%)	465 (14.2%)	632 (19.3%)	481 (14.6%)	453 (13.8%)	350 (10.7%)	10 (0.3%)	3.9 ± 1.9
Online learning should play a more prominent role in medical education	289 (8.8%)	290 (8.8%)	312 (9.5%)	501 (15.2%)	518 (15.8%)	589 (17.9%)	780 (23.7%)	7 (0.2%)	4.7 ± 1.9
Medical education is lagging behind in online learning	49 (1.5%)	83 (2.5%)	140 (4.3%)	449 (13.7%)	711 (21.6%)	1088 (33.1%)	752 (22.9%)	14 (0.4%)	5.4 ± 1.4
Online learning can harmonize the curricula	166 (5.1%)	354 (10.8%)	368 (11.2%)	875 (26.6%)	632 (19.2%)	613 (18.7%)	210 (6.4%)	68 (2.1%)	4.3 ± 1.6
I expect lecturers to be familiar with online learning	28 (0.9%)	79 (2.4%)	120 (3.6%)	324 (9.9%)	697 (21.2%)	1211 (36.9%)	812 (24.7%)	15 (0.5%)	5.6 ± 1.3
Lecturers have sufficient previous experience in online learning	320 (9.7%)	697 (21.2%)	723 (22.0%)	815 (24.8%)	457 (13.9%)	220 (6.7%)	42 (1.3%)	12 (0.4%)	3.4 ± 1.4
Switching to online courses led to a higher participation in courses	231 (7.0%)	342 (10.4%)	377 (11.5%)	941 (28.6%)	446 (13.6%)	517 (15.7%)	390 (11.9%)	42 (1.3%)	4.3 ± 1.7

SD=standard deviation; N/A=not available
