# Supplementary material for: How COVID-19 kick-started online learning in medical education—The DigiMed study
Source: PLoS One. 2021 Sep 21;16(9):e0257394. doi: 10.1371/journal.pone.0257394 (PMC8454930; doi:10.1371/journal.pone.0257394)
Supplement: S5 Table — (PDF) [file pone.0257394.s008.pdf]

S8 Table. Subgroup analysis regarding study year

Statement	Mean value (year 1-3)	Mean value (year 4-6)	Difference between mean values	P value	Cohen´s d	Effect size
Since the pandemic, my medical school successfully switched to online courses	5.1	5.4	0.3	<0.01	0.214	small
Before the pandemic, my medical school already offered many online courses	2.7	2.9	0.2	<0.01	0.134	negligible
I am happy with the quantity of online courses provided	4.8	5.0	0.2	<0.01	0.175	negligible
I am happy with the quality of online courses provided	4.6	4.7	0.1	<0.01	0.095	negligible
I regularly use social media	5.6	5.6	0	0.53	-0.022	negligible
I have the devices required for online learning	6.6	6.5	0.1	0.03	-0.079	negligible
I think it is acceptable to own the devices required for online learning	5.4	5.3	0.1	0.01	-0.097	negligible
I feel comfortable using the software required for online learning	5.6	5.4	0.2	0.01	-0.092	negligible
I feel well prepared for online learning	5.5	5.2	0.3	<0.01	-0.221	negligible
Online courses give me a greater flexibility	5.9	5.6	0.3	<0.01	-0.246	small
I find it difficult to motivate myself to follow	3.7	4.1	0.4	<0.01	0.238	negligible
Online learning bears the risk of social isolation	4.7	5.0	0.3	<0.01	0.208	small
Online learning offers sufficient possibilities to interact with my fellow students	3.0	3.0	0	0.50	-0.023	negligible
I am concerned about my privacy when using online learning	2.5	2.7	0.2	<0.01	0.129	negligible
Online learning offers sufficient possibilities to interact with the lecturer	4.2	3.9	0.3	<0.01	-0.180	negligible
Online learning increases the quality of medical education	4.3	3.6	0.6	<0.01	-0.355	small
Online learning should play a more prominent role in medical education	4.6	5.0	0.4	<0.01	-0.248	small
Medical education is lagging behind in online learning	5.7	5.3	0.4	<0.01	-0.282	small
Online learning can harmonize the curricula	4.5	4.1	0.4	<0.01	-0.252	small
I expect lecturers to be familiar with online learning	5.7	5.5	0.2	<0.01	-0.158	negligible
Lecturers have sufficient previous experience in online learning	3.2	3.5	0.3	<0.01	0.247	small
Switching to online courses led to a higher participation in courses	4.4	4.2	0.2	<0.01	-0.135	negligible
