# Supplementary material for: How COVID-19 kick-started online learning in medical education—The DigiMed study
Source: PLoS One. 2021 Sep 21;16(9):e0257394. doi: 10.1371/journal.pone.0257394 (PMC8454930; doi:10.1371/journal.pone.0257394)
Supplement: S6 Table — (PDF) [file pone.0257394.s009.pdf]

S9 Table. Subgroup analysis regarding gender

Statement	Mean value (female)	Mean value (male)	Difference between mean values	P value	Cohen´s d	Effect size
Since the pandemic, my medical school successfully switched to online courses	5.3	5.2	0.1	0.06	0.073	negligible
Before the pandemic, my medical school already offered many online courses	2.8	2.8	0	0.78	-0.010	negligible
I am happy with the quantity of online courses provided	5.1	4.9	0.2	0.04	0.099	negligible
I am happy with the quality of online courses provided	5.0	4.6	0.4	<0.01	0.174	negligible
I regularly use social media	5.7	4.8	0.9	0.02	0.088	negligible
I have the devices required for online learning	6.6	6.6	0	0.01	-0.095	negligible
I think it is acceptable to own the devices required for online learning	5.2	5.5	0.3	<0.01	-0.178	negligible
I feel comfortable using the software required for online learning	5.4	5.8	0.4	<0.01	-0.256	small
I feel well prepared for online learning	5.2	5.5	0.3	<0.01	-0.176	negligible
Online courses give me a greater flexibility	5.8	5.7	0.1	0.04	0.077	negligible
I find it difficult to motivate myself to follow	3.9	4.1	0.2	0.03	-0.082	negligible
Online learning bears the risk of social isolation	4.9	4.8	0.1	0.18	0.049	negligible
Online learning offers sufficient possibilities to interact with my fellow students	3.0	3.0	0	0.58	-0.021	negligible
I am concerned about my privacy when using online learning	2.6	2.6	0	0.67	0.016	negligible
Online learning offers sufficient possibilities to interact with the lecturer	4.2	4.1	0.1	0.14	-0.054	negligible
Online learning increases the quality of medical education	3.9	3.9	0	0.93	-0.003	negligible
Online learning should play a more prominent role in medical education	4.7	4.8	0.1	0.53	-0.030	negligible
Medical education is lagging behind in online learning	5.4	5.4	0	0.59	0.020	negligible
Online learning can harmonize the curricula	4.3	4.3	0	0.32	-0.037	negligible
I expect lecturers to be familiar with online learning	5.6	5.6	0	0.09	-0.063	negligible
Lecturers have sufficient previous experience in online learning	3.4	3.4	0	0.67	0.016	negligible
Switching to online courses led to a higher participation in courses	4.3	4.2	0.1	0.01	0.106	negligible
